# Sex Differences in Racing History of Recreational 10 km to Ultra Runners (Part B)—Results from the NURMI Study (Step 2)

**DOI:** 10.3390/ijerph192013291

**Published:** 2022-10-14

**Authors:** Mohamad Motevalli, Derrick Tanous, Gerold Wirnitzer, Claus Leitzmann, Thomas Rosemann, Beat Knechtle, Katharina Wirnitzer

**Affiliations:** 1 Department of Sport Science, Leopold-Franzens University of Innsbruck, 6020 Innsbruck, Austria; 2 Department of Research and Development in Teacher Education, University College of Teacher Education, Tyrol, 6010 Innsbruck, Austria; 3 adventureV & change2V, 6135 Stans, Austria; 4 Institute of Nutrition, University of Gießen, 35390 Gießen, Germany; 5 Institute of Primary Care, University of Zurich, 8000 Zurich, Switzerland; 6 Medbase St. Gallen Am Vadianplatz, 9000 St. Gallen, Switzerland; 7 Research Center Medical Humanities, Leopold-Franzens University of Innsbruck, 6020 Innsbruck, Austria

**Keywords:** running, marathon, gender, female, competition, performance, behavior, habit, endurance

## Abstract

Sex differences in anatomy and physiology are the primary underlying factor for distinctions in running performance. Overall participation in recreational running events has been dominated by males, although increasing female participation has been reported in recent years. The NURMI study participants filled in a survey following the cross-sectional study design with questions on sociodemographic data, running and racing motivations, training behaviors, and racing history and experience. Data analysis included 141 female and 104 male participants aged 39 (IQR 17) with a healthy median BMI (21.7 kg/m²; IQR 3.5). Statistical analyses revealed sex differences with the males performing faster at half-marathon (*p* < 0.001) and marathon (*p* < 0.001) events but no difference at ultra-marathons (*p* = 0.760). Mediation analyses revealed no significant sex differences in the performance of half-marathon and marathon when considering training behaviors (*p* > 0.05), racing history (*p* > 0.05), or racing experience (*p* > 0.05). Differences in recreational performance may be more closely related to social constraints and expectations of females rather than the physiological advantages of the male athlete. Health professionals who guide and support recreational runners as well as the runners themselves and their coaches may benefit from this study’s results in order to improve the best time performance through a deeper understanding of the areas that mediate sex differences.

## 1. Introduction

Previous research has identified a clear sex-based difference in running performance [1,2,3], which underlies the separation of sexes at running competitions [4], although this difference has been reported to diminish with distances beyond that of a marathon [5,6]. Runner training and preparation, however, appear to be crucial for racing performance regardless of sex [7]. To date, several studies have investigated the sex-based differences in the performance of recreational runners of specific distances [8,9,10], but to the best of the authors knowledge, no study has assessed sex-specific differences in recreational 10-kilometer (10 km), half-marathon (HM), marathon (M), and ultra-marathon (UM) runners in a single analysis.

Anatomical (e.g., overall body size and weight, anthropometrics, body composition) [2,8] and physiological sex differences (e.g., aerobic capacity, muscular strength) [11] are reported to contribute to an advantage for the male runner. Thus, a biological advantage in favor of the male runner in the best time marathon performance of approximately 9-11% has been reported [12,13,14] and appears to be due to various factors related to sex (e.g., greater body fat percentage and proportion of extremity vs. trunk fat in females, smaller hearts among females, and lower serum iron among females), potentially leading to a greater submaximal volume of oxygen consumption, increased lactate levels, and a higher heart rate when gradually increasing the running workload [1,11,12,15]. However, social circumstances may unnecessarily hinder female performance [11]. For example, overall running event participation has been dominantly male [16]. In addition, the antiquated biases that were once widely accepted in most developed countries in the 1960s may still linger across societies today regarding women’s athletic abilities, such as that long-distance endurance running is unhealthy and even harmful to the female anatomy [16]. Nowadays, running participation is reported as healthy for both sexes based on conclusive, detailed analyses [17,18,19,20]. Despite the previous misconceptions, women’s participation in running races has been steadily increasing to nearly match that of men today [21].

In addition to health [19,20,22], running event participants are often oriented towards racing through leisure or performance motivations [23,24], and participating in distance running events (10 km, HM, M, or UM) provides an opportunity for focusing on personal goals. The racing environment, the season of event, as well as individual characteristics such as anthropometrics, personality and motivations, nutritional status, and racing history/experience all contribute to the best time performances [7,25,26]. Therefore, the objective of this study is to analyze sex-related differences in racing history, experience, and performance of recreational 10 km to ultra-distance runners for the first time and whether training behaviors, racing history, or racing experience mediate a potential performance relationship. As there seems to be contradictions and inconsistency of results regarding the differences and similarities in endurance runners between the sexes in the available literature, especially based on the previous literature [8,9,10], this investigation hypothesizes there is a difference in best time race performance between the sexes of recreational distance runners.

## 2. Materials and Methods

The profile of the present investigation methodology has been described previously (see Part A of the sequenced paper) [27] and detailed information for interested readers are available elsewhere [22,28]. In summary, the Nutrition and Running High Mileage (NURMI) study followed a protocol [29] approved by the ethics board of St. Gallen, Switzerland on 6 May 2015 (EKSG 14/145) and has the trial registration number of ISRCTN73074080, which was registered retrospectively. The subjects, who were distance runners competing over 10 km to UM, were required to provide informed consent prior to participating in the NURMI study. Then, participants completed an online survey containing questions on sociodemographic information and a complete profile of running- and performance-related data.

**Figure 1:**
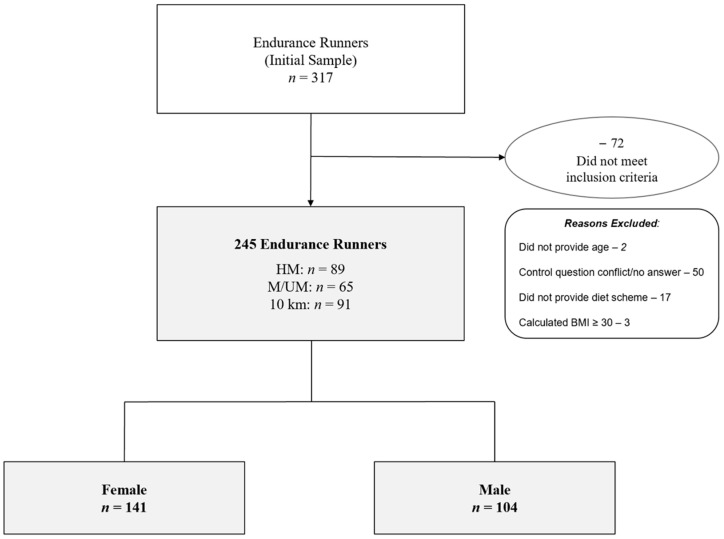
Flow Chart of Participants’ Enrollment by Race Distance and Sex. BMI—body mass index. HM—half-marathon. M/UM—marathon/ultra-marathon. 10 km—10 kilometers.

The participants characteristics are presented in Table 1. The interested reader is kindly referred to the Part A publication for the participants’ recruitment and study procedures [27]. Figure 1 shows the enrollment and categorization of participants.

The race performances and training behaviors of active female and male distance runners were described using the subsequential details: total and respective number of completed running events (HM, M/UM, 10 km); number of years of active running completed without break; racing history (age at first race; first race distance: 10 km, HM, M, UM; successful completion of HM, M, UM in last two years; ratio of completed HM/M to other events; best HM time, best M time, best UM time), running training (frequency of training, and daily and weekly span of training (hours, km) related to periodized phases and stages of training, respectively) and preparation for the main event (professional training resource; total duration). Running performance was linked to a normalized aggregate mean considering the best finishing time of HM and M and transformed to an index range (0–100).

R software (version 3.6.2 Core Team 2019: R Foundation for Statistical Computing, Vienna, Austria) was used to perform all the statistical analyses. Descriptive statistics (median, interquartile range (IQR); mean, standard deviation (SD)) were used for the exploratory analysis. PCA was used in identifying the two latent factors.

Significant differences in racing activity (history, training, racing, etc.) between race distance and sex were calculated with a non-parametric test. Associations between variables were performed by Chi-square test (χ²; nominal scale) and Wilcoxon test (ordinal and metric scale) and were approximated by using the F distributions with ordinary least squares.

Multiple linear regression analysis and multivariate linear regression were used to test differences in the performance, health, and leisure motivations of female and male runners. The results of the regression are displayed as effect plots (95% confidence interval (95%-CI)).

The level of statistical significance was set at *p* ≤ 0.05.

## 3. Results

A total of 317 distance runners completed the questionnaire. Following data clearance, 72 participants did not meet the inclusion criteria; therefore, 245 runners (141 female, and 104 male) made up the final sample with a median body weight of 65 kg (IQR 14.2), height of 1.7 m (IQR 0.01), BMI of 21.7 kg/m² (IQR 3.5), and age of 39 (IQR 17) years. The runners resided in various countries such as Germany (72%), Austria (18%), Switzerland (5%), or other (4%).

There were significant differences between the sexes regarding body weight (*p* < 0.001), height (*p* < 0.001), and BMI (*p* < 0.001), with the males being heavier (73 kg, IQR 11.9), taller (1.8 m, IQR 0.1), and having a higher BMI (22.8, IQR 3.16). Moreover, 15 (67% female) participants were divorced (or separated), 164 (52% female) were married (or living with partner) and 66 (68% female) were single. Concerning the participants’ educational background, there were 44 females (vs. 39 males) with a high school degree or equivalent, 49 females (vs. 34 males) with a graduate degree (university level), and 31 females (vs. 22 males) with A-Level or equivalent; one female had no degree, while a total of 25 runners provided no answer. A significant sex difference was found regarding exercise focus (*p* = 0.044), where 81 females (vs. 52 males) were leisure focused, with more males (44%; *n* = 46) focused on sports performance (30%) compared to females (30%; *n* = 43). A total of 154 participants were included as NURMI-runners racing at the HM or M/UM distance, including a greater proportion of males (52% male), and an additional 91 participants (26% male) were 10 km runners (*p* < 0.001). The males had completed more 10 km (8, IQR 15), HM (10, IQR 15), and M/UM (10, IQR 11) races than females (6, IQR 7; 6, IQR 6; 10, IQR 10, respectively). Characteristics of participants with racing motivation, history, and preferences of females and males, including racing experience, are shown in Table 1. Further details on the profile of the total sample and the sex-specific subsamples are provided in Part A [27].

**Table 1:** Distance Runner Characteristics, including Racing Motivation, Preferences, and Experience Displayed by Sex.

		Total	Female	Male	Statistics
		100% (245)	58% (141)	42% (104)	
**Age (years)**		39 (IQR 17)	37 (IQR 16)	43 (IQR 18)	F_(1, 243)_ = 7.03 *p* = 0.009
**BMI (kg/m²)**		21.7 (IQR 3.5)	20.9 (IQR 3.01)	22.8 (IQR 3.16)	F_(1, 243)_ = 28.72 *p* < 0.001
**Marital Status**	Divorced/Separated	6% (15)	7% (10)	5% (5)	χ^2^_(2)_ = 5.32 *p* = 0.70
	Married/With Partner	67% 164	61% (86)	75% (78)	
	Single	27% (66)	32% (45)	20% (21)	
**Racing Distance**	10 km	37% (91)	48% (67)	23% (24)	χ^2^_(2)_ = 19.55 *p* < 0.001
	HM	36% (89)	35% (49)	38% (40)	
	M/UM	27% (65)	18% (25)	38% (40)	
**Racing Motivation**	Leisure	46% (106)	50% (65)	41% (41)	χ^2^_(1)_ = 1.70 *p* = 0.193
	Sport Performance	54% (125)	50% (66)	59% (59)	
**Favorite Race Season**	Winter	<1% (2)	2% (2)	/	χ^2^_(3)_ = 4.45 *p* = 0.216
	Spring	46% (106)	47% (62)	44% (44)	
	Summer	23% (52)	18% (24)	28% (28)	
	Autumn	31% (71)	33% (43)	28% (28)	
**Years Active in Running**		7 (IQR 7)	5 (IQR 7)	8 (IQR 11)	F_(1, 242)_ = 10.75 *p* = 0.001
**First Race Age (years)**	10 km	30 (IQ 16)	31 (IQR 16)	29 (IQR 15)	F_(1, 152)_ = 1.62 *p* = 0.205
	HM	32 (IQR 16)	33 (IQR 15)	32 (IQR 16)	F_(1, 217)_ = 0.13 *p* = 0.720
	M	35 (IQR 13)	35 (IQR 14)	34 (IQR 12)	F_(1, 136)_ = 0.38 *p* = 0.539
	UM	40 (IQR 11)	41 (IQR 8)	38 (IQR 12)	F_(1, 46)_ = 0.75 *p* = 0.391
	Total	30 (IQR 16)	31 (IQR 16)	30 (IQR 18)	F_(1, 240)_ = 0.05 *p* = 0.831
**First Race Distance**	10 km	65% (157)	70% (97)	58% (60)	χ^2^_(2)_ = 4.21 *p* = 0.122
	HM	27% (65)	24% (33)	31% (32)	
	M	9% (21)	6% (9)	12% (12)	
**Races Completed in Total**		8 (IQR 11)	7 (IQR 9)	10 (IQR 13)	F_(1, 243)_ = 6.75 *p* = 0.010
**Ratio of HM/M to Other Completed Events**		40 (IQR 50)	40 (IQR 51)	40 (IQR 48)	F_(1, 243)_ = 0.07 *p* = 0.791
**Best Finishing Time (minutes)**	HM	111 ± 33	122 ± 39	98 ± 14	F_(1, 215)_ = 72.41 *p* < 0.001
	M	230 ± 45	252 ± 49	213 ± 32	F_(1, 130)_ = 28.57 *p* < 0.001
	UM	628 ± 489	662 ± 579	614 ± 454	F_(1, 44)_ = 0.09 *p* = 0.760

Note. Results are presented as percentage (%), total numbers, median (IQR), and mean ± SD. χ^2^ statistic calculated by Pearson’s Chi-squared test and *F* statistic calculated by Wilxocon test. 10 km—10 kilometers. HM—half-marathon. M/UM—marathon/ultra-marathon.

No significant differences were found between the sexes in the motivation for racing (*p* = 0.193) or the current running motivation (*p* = 0.356). The favored season for running events did not differ between females and males (*p* = 0.216), participants most frequently preferred racing (*n* = 106; 42% male) in the springtime. Significant differences were found between the sexes racing history based on: (i) years fully active in running (*p* = 0.001), where males finished more complete years of running (8, IQR 11) than females (5, IQR 7); (ii) total races completed (*p* = 0.010), where males finished more events (10, IQR 13) than females (7, IQR 9); (iii) best finishing time over the HM distance (*p* < 0.001), in which males were faster on average (98 ± 14 min) compared to females (122 ± 39 min); (iv) best finishing time over the M distance (*p* < 0.001), in which males were faster on average (213 ± 32 min) compared to females (252 ± 49 min); (v) successful completion of planned M events over the previous two years (*p* < 0.001), where males finished more events (1, IQR 3) than females (0, IQR 1); and (vi) successful completion of planned UM events over the last two years (*p* < 0.001), where males finished more events (0, IQR 1) than females (0, IQR 0). No significant differences were observed for racing experience/history considering the age at the first time participating in a running event (*p* = 0.831), also for each race distance separately (10 km, HM, M, UM; *p* > 0.05), the first event race distance–whether 10 km, HM, M/UM—(*p* = 0.122), the best finishing time over the UM distance (females 662 ± 579 min vs. males 614 ± 454 min; *p* = 0.760), or the successful completion of planned HM events (*p* = 0.728). 

**Figure 2:**
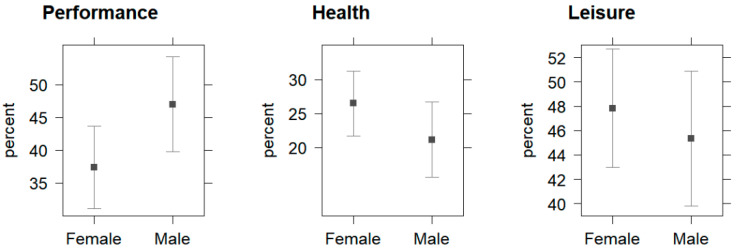
Effect plots with 95%-CI displaying the differences between female and male running exercise/racing motives (*n* = 231): performance, health, and leisure. Note. 95%-CIs were computed using the multivariate regression analyses (Wald approximation).

A sex difference was identified by a multivariate linear regression, with the male runners being significantly more sport performance motivated than the females (b = 9.6; 95% CI [0.02–19.2]; *p* < 0.05). No sex differences in health (b = −5.46; 95% CI [−12.9–1.93]; *p* > 0.05) or leisure motives (b = −2.5; 95% CI [−9.87–4.86]; *p* > 0.05) were identified by multivariate linear regression, as displayed by Figure 2.

Multivariate linear regression analyses were used to predict the best HM and M running time performance based on the following confounders: (i) the participants’ sex and training behavior–preparatory stage 3 (weekly training frequency, mileage and hours, and daily training mileage and hours), preparatory stage 4 (weekly training frequency, mileage and hours, and daily training mileage and hours), weekly mileage of preparatory stage 1, professional advice sought, and pre-event training duration, which explained 22% of the variance (adjusted R^2^ = 0.22) with no significant difference found for sex (b = −0.295; 95% CI [−7.7–7.11]; *p* > 0.05); (ii) the participants’ sex, and racing history (years fully active in running and age at first running event), in which the model explains 16% of the variance (adjusted R^2^ = 0.16), and no significant difference was found for sex (b = −1.46; 95% CI [−8.93–6]; *p* > 0.05); and (iii) the participants’ sex and racing experience (HM races completed, M races completed, the ratio of HM/M to total events completed, and total events completed), which explains 14% of the variance (adjusted R^2^ = 0.14) with no significant difference found for sex (b = −1.72; 95% CI [−9.14–5.7]; *p* > 0.05).

## 4. Discussion

This study aimed to identify sex differences in racing history, experience, and race performance among recreational 10 KM to UM distance runners. The most important findings were (1) males weighed more, were taller, and had a higher BMI than female runners; (2) there was no sex difference in racing motivation; however, when the running motivations (initial, current, racing) were combined with exercise focus, a significant sex difference was identified, with males being significantly more sport performance motivated; (3) males accumulated significantly more years of active running and completed significantly more races, especially for marathons and ultra-marathons within the last 2 years; (4) a significant sex difference in best time performance with males being faster on average at HM and M distances. Therefore, this investigation verifies the initial hypothesis that there is a sex difference in racing best time performance of recreational distance runners with males being significantly faster on average at HM and M events. (5) However, when considering best time performance as an index and including various mediators (training behavior, racing history, or racing experience) within multivariate linear regression models, no significant sex differences in performance were found.

The findings of the anthropometric proportions of this study’s participants (weight, height, BMI) are consistent with previous reports [8], indicating that males have generally larger bodies and thus greater body weight than females. Females normally have a greater body fat percentage than males [30], and this has been reported to be a factor limiting long-distance running performance [1]. Excess weight, in particular, likely contributes to a disadvantage for the long-distance runner’s best time performance independent of sex, considering that each additional gram must be actively transported, resulting in greater energy expenditure [11,20,31]. Although possible participants with obesity were excluded from this investigation based on the WHO criteria [32,33], the male runners (73 kg) remained significantly heavier than the females (59.5 kg). Likewise, the male BMI was significantly larger (+1.9 kg/m^2^) within the present sample. However, the BMI calculation does not take body composition into account [32,33], excluding potential confounders such as skeletal and lean body mass regarding the classification of individuals [34]. Moreover, the present sample is based on runners of predominantly long (HM) or very long (M/UM) distances who are well known to measure around the lower boundaries of body fat percentage compared to less active populations [35,36], which could indicate a greater skeletal and lean body mass among the males that is known to positively affect performance [11,20,31,34]. Indeed, reports of female runners developing eating disorders due to a strategy to minimize body fat are not uncommon [20,37], which is linked to malnourishment and the co-occurrence of menstrual dysfunction and osteopenia [20,37]. However, this outcome may be limited only to sport performance motivated females [22]. Thus, there may be less of a psychological burden for male runners concerning body composition, which likely transfers to a higher level of psychological well-being, especially for performing in racing events [38].

Regarding the finding that motivations for racing were similar between the sexes, previous research has found males to be more sport performance oriented [39]. However, within the regression model, including all of the motives as covariates (initial running, exercise, current running, racing), the male dominance in sports performance motivation was in line with previous research [39]. This finding may be related to the fact that males generally tend to be more competitive than females, especially in athletics [40]. In addition, the present study found that the males had completed significantly more years fully active in running than their female counterparts, which is also consistent with previous findings [41]; however, this may be due to the fact that the males were significantly older in this sample. Likewise, the males had completed significantly more events, which shows that they are more dedicated and established in running and finishing races, possibly due to their greater level of focus on competition and sport performance and age experience [39,40]. In turn, this finding is likely also related to the successful completion of marathon and ultra-marathon events within the previous two years, where the males finished significantly more long-distance events than the females. However, there were significantly more males within the present sample racing at the M/UM distances, which appears to contradict previous reports of increasing female participation in distance races [21]. Although, it could be possible that more females were participating in distance events (HM, M/UM) within this sample but had not successfully completed an event to date, which would have led to their exclusion from the present analyses. Interestingly, runners of extreme distances (UM) have been reported to separate from their partners or divorce due to a lack of support in running, considering the high amount of time spent training and performing in events [42]. Regarding the marital status of the present runner sample, no sex differences were identified even though more males were racing at the UM distance (40 males vs. 25 females).

It was found that male runners performed significantly better than the females in HM and M events, considering their best times in which the males were 24 min and 39 min faster on average, respectively. This finding is consistent with the previous literature comparing sex differences in performance of recreational runners that show a 10% difference at the minimum [43]. However, it is interesting to find that there was no sex-based performance difference in the best time to complete an ultra-marathon, but this analysis did not take into account the exact UM distance, which could have been any distance ≥50 km. Therefore, this result most likely suggests that the males were racing at longer UM distances than the females [2,3,5,6]. Furthermore, when controlling the best HM and M runtimes with the basic underlying factor of training behavior (including training duration, professional support, and specific variables within the main training preparatory period), no performance difference remained between the sexes. Previously, when sex differences in performances of elite runners were analyzed, males have been found to be faster regardless of matched training [12], and the present data on recreational runners appear to contradict that, possibly due to a more heterogeneous study sample in terms of performance ability (e.g., anthropometrics, skeletal muscle fiber composition) [13].

In addition, another multivariate linear regression model, including the covariates of racing history (age at first running event), indicated no sex difference in HM and M best time performance. This finding suggests that the males had a more robust background in racing and therefore running, which is inconsistent among other reports [8,44], but is reflected by the males having a significantly greater age than the females in this study. The connection to best time performance may be a result of long-term running adaptations due to a comparable involvement in completing the overarching periodization scheme for each sex, including main competitions over the years [8,45].

Lastly, a third multivariate linear regression model based on racing experience (number of completed HM and M events and the proportion of total events completed) also showed no sex difference in HM and M best time performance. Thus, being successful in completing running events is an important contributor for best time performance, regardless of sex, as seen previously [46]. Therefore, considering the best time performances of distance runners, males are generally faster; however, this sex difference was superficial in recreational distance runners when considering several modifiable areas that mediate performance, especially the training behaviors within Phase 2: the main preparatory period, considering that this model explained 22% of the variance (vs. racing history 16% vs. racing experience 14%). These findings may indicate that physiological sex differences are negligent regarding HM and M performance in recreational distance runners, and that social circumstances may play a considerable role in best time running performance.

Based on the cross-sectional design of the study, the findings of the present investigation include limitations that must be considered, such as that no causative conclusions can be drawn from these results. The primary limitation to be addressed is that self-report is a common feature of the questionnaire approach that is well-known to result in a misrepresentation of answers, likely due to social views. As a means to limit the effect of misreporting, control questions were included throughout the survey, and, likewise, the participants were highly motivated distance runners, which may have enhanced the reliability of their answers and the dataset. In addition, while 245 distance runners were included within the final analysis, the sample size was relatively modest, and the race distances were unevenly distributed, including more males in the longer distances (M/UM). Moreover, several factors of race events were not considered in the analyses, such as the race environment and conditions (inclement or mild weather along with temperature and relative humidity), time of day, season, and the event location; however, the best time reports were verified retrospectively. Another limitation of the present investigation is that nutritional status was not controlled for, or the type of nutrition followed by the runners, which is a well-known factor affecting performance [25,26,29]. However, the NURMI study has included participant nutritional data that have or will be reported in subsequent papers due to publication requirements.

Considering the limitations of this investigation, valuable insights may be taken by female and male recreational distance running athletes, as well as their coaches, athletic trainers, exercise physiologists, physical therapists, and physicians, regarding essential factors contributing to a healthy best performance at running events. Future studies are suggested to investigate sex differences of recreational runners of long distances (HM, M, UM) in best time performance considering the main underlying focus of sport performance and controlling for mediators of running and racing history, professional support, and racing experience.

## 5. Conclusions

This study was the first with the aim of investigating sex-associated differences in racing history, experience, and performance of recreational 10 km, HM, M, and UM distance runners. The findings of the present study reveal males are faster in their best time performances at HM and M events and report a more robust background in running and racing history and experience. However, recreational female distance runners compete comparably with males considering the best time performance of HM and M events when statistically controlling for training behaviors or similar backgrounds in racing experience/history. Thus, partaking in and, most importantly, finishing running events is key in improving best time performances for females and males alike. The results of this investigation provide a critical insight into the crucial differences in female and male recreational distance runner performance over several long distances (HM, M/UM, 10 km), which may be necessary for lifting the current societal circumstances (training/racing opportunities, performance expectations) and understandings of health and exercise professionals (athletic trainers, exercise physiologists, sports medicine doctors, physical therapists) in supporting or limiting female participation. Moreover, competitive runners, health professionals and coaches who supervise and counsel recreational runners may benefit from these findings in order to improve best time performance through a deeper understanding of the areas that mediate sex differences.

## Data Availability

The data sets generated during and/or analyzed during the current study are not publicly available but may be made available upon reasonable request. Subjects will receive a brief summary of the results of the NURMI Study if desired.
